# Salmonellosis: Serotypes, prevalence and multi-drug resistant profiles of *Salmonella enterica* in selected poultry farms, Kwara State, North Central Nigeria

**DOI:** 10.4102/ojvr.v86i1.1667

**Published:** 2019-05-30

**Authors:** Akeem O. Ahmed, Moshood A. Raji, Paul H. Mamman, Clara N. Kwanashie, Ibrahim A. Raufu, Abdulfatai Aremu, Ganiu J. Akorede

**Affiliations:** 1 Department of Veterinary Microbiology, University of Ilorin, Ilorin, Nigeria; 2 Department of Veterinary Microbiology, Ahmadu Bello University, Zaria, Nigeria; 3 Department of Veterinary Pharmacology and Toxicology, University of Ilorin, Ilorin, Nigeria

**Keywords:** Nigeria, antimicrobials, fluoroquinolone, *Salmonella Agama*, poultry environment, Kwara

## Abstract

Salmonellosis is a major threat facing the poultry industry globally. This study was conducted to investigate the level of *Salmonella* contaminations and determine the resistance pattern of isolates obtained from selected poultry farms in Kwara State, a transition state between southern and northern regions of Nigeria. A total of 900 samples were collected between January and August 2017, from the poultry environment, apparently including healthy and dead birds. *Salmonella* was isolated and identified using standard bacteriological methods. All presumptive *Salmonella* isolates were serotyped and tested for antimicrobial susceptibility using 11 different antimicrobials. A total of 58 (6.4%) *Salmonella* isolates were obtained, and the isolation rate was only statistically significant (*p* < 0.05) in live birds. The isolates comprised of 13 serovars. The three predominant serovars, *Salmonella enterica* ser. 6.7:d:- (29.0%), *Salmonella* Agama (28.0%) and *Salmonella* Typhimurium (16.0%), were isolated from all three sample types. Rare serovars like *Salmonella* Albany, *Salmonella* Colindale, *Salmonella* Istanbul, *Salmonella* Larochelle, *Salmonella* Nigeria and *Salmonella* Orion were also isolated in this study. A high frequency of resistance was generally observed with all the isolates exhibiting a total of (100%) resistance to ampicillin, cefotaxime and ceftazidime. This study documents the first predominant isolation of *S. enterica* ser. 6.7:d:- and *S.* Agama from chickens. It also documents the high frequency of fluoroquinolone and cephalosporins resistance of the isolates indicating the presence of selective pressure in the environment. Controls and targeted interventions against *Salmonella* and the frequent occurrence of antimicrobial resistance in chickens should be initiated to prevent the spread of this organism.

## Introduction

Salmonellosis in poultry is endemic worldwide, causing morbidity and mortality and, thus, economic losses (Abiodun et al. 2014; Ahmed et al. 2017; Akter et al. 2007; Kwon et al. 2010). The disease is very significant by virtue of the fact that *Salmonella* can be transmitted vertically from parent to offspring. The control of salmonellosis in the poultry industry is complicated because, in addition to vertical transmission from parent stock to offspring, horizontal transmission on farms is also common; this makes its control a challenge (Abiodun et al. 2014; Dawoud et al. 2011; Hannah et al. 2011). Poultry can become infected by the horizontal route via infected litter, faeces, feed, water, dust, fluff insects, equipment, fomites, diseased chicks and rodents, contaminated with *Salmonella* (Poppe 2000). They can also be transmitted by other animals, wild birds and personnel. *Salmonella* may contaminate young chicks directly through ovarian transmission or penetrate the egg shell after the egg has been laid (Cox, Berrang & Cason 2000; Maryam et al. 2009). Poultry farms and poultry products are the major sources for *Salmonella* contamination (Hussein, Hala & Khalil 2009). Reports on various poultry diseases occurring in some parts of this country showed that salmonellosis is the major threat facing poultry production in Nigeria (Mamman et al. 2014), and animal droppings have been shown to be a potential reservoir for many enteric organisms (Raufu et al. 2013). Hence, consumers of poultry and poultry products are at risk of contracting salmonellosis via consumption of contaminated products (Adesiyun et al. 2005; Mughini-Gras et al. 2014).

Although vaccination to prevent salmonellosis has been practised successfully on layer farms in several countries (Dawoud et al. 2011; Kwon et al. 2010), vaccines produced from local isolates are still not readily available on the market, especially in developing countries, for effective preventive measures. Hence, the control of salmonellosis predominantly lies on good sanitary practices and the use of antimicrobial drugs for prophylaxis and therapeutics (Abiodun et al. 2014; Akter et al. 2007), which subsequently leads to abuse of antimicrobial drugs in poultry settings, culminating in the development of resistance and the eventual limitation of the therapeutic outcome in the treatment of bacterial diseases (Cantas et al. 2013; Sasanya et al. 2005).

It is usually difficult to report the occurrence of salmonellosis and antimicrobial resistance in developing countries like Nigeria because of a lack of coordinated surveillance systems. Studies so far in Nigeria have only included a limited number of samples or isolates from a single or a few reservoirs and limited geographical coverage (Akinyemi et al. 2010; Fashae et al. 2010; Orji, Onuigbo & Mbata 2005; Raufu et al. 2013).

The purpose of this study was to determine the level of *Salmonella* contamination and the frequency of antimicrobial resistance in the isolates obtained from intensively managed poultry farms in Kwara State, North Central Nigeria.

## Materials and methods

### Study area

**FIGURE 1:**
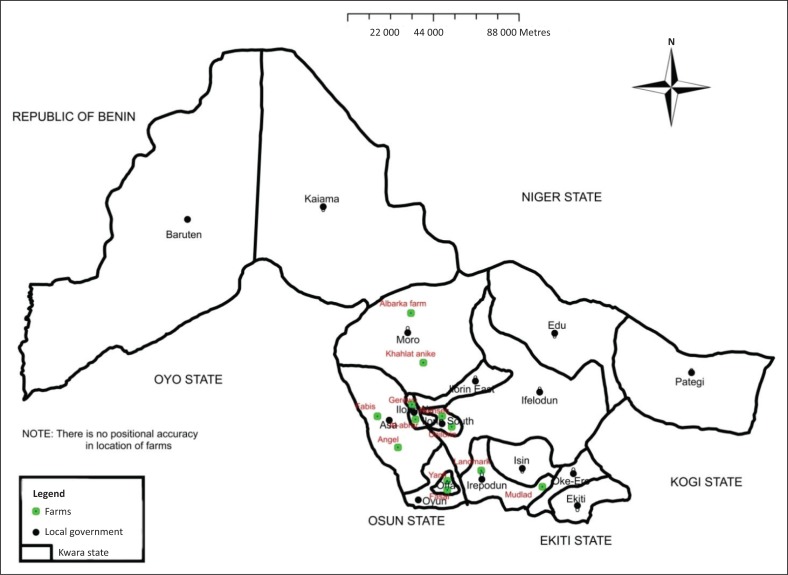
A map of Kwara State showing locations of the sampling sites within selected local government areas.

The study was conducted in Kwara State, North Central Nigeria, from January to August 2017. Kwara State has 16 local government areas, and this study was conducted in the local government areas with well-established commercial poultry farms. Twelve farms were randomly selected, out of which only nine consented to participate and three declined. All the farms sampled were registered with the Kwara State Veterinary Services (Figure 1).

### Sample types and collection

A total of 900 samples (100 samples/farm) were collected. Each farm was visited at least three times and the following samples were collected: cloacal swabs, environmental swabs of deep litters, poultry feed, water and organs (liver, spleen, ovarian follicle, caecum and heart) all from recently dead birds as shown in Table 1.

**TABLE 1:** Types and number of samples.

Source	Samples	
	Types	No. (per farm)
Dead birds	Liver	8
	Spleen	8
	Heart	8
	Ovarian follicle	8
	Caecum	8
Live birds	Cloacal swab	35
Poultry environment	Feed from feeder	5
	Feed from feed store	5
	Water from drinker	5
	Water from water tank	5
	Litters swabs	5
**Total**	**-**	**100**

The procedures used for sample collection and transportation to the laboratory were in accordance with the method described previously (Mamman et al. 2014). Cloacal samples were collected using sterile swabs; these were inserted into the cloaca of randomly selected birds and rotated inside the cloaca. Samples of organs from dead birds were collected by aseptically opening a freshly dead bird and picking approximately 5 grams (g) of the required organ (one organ per bird); 5 g of litters, approximately 10 g of feed each from a feed store and feeding trough, and 10 mL each of untreated water from a water tank and drinkers were collected from each farm.

### Isolation and identification of bacteria

The samples were analysed at the Veterinary Microbiology Laboratory of the Faculty of Veterinary Medicine, University of Ilorin, Nigeria, within 24 hours (h) of collection according to the method previously described by Ahmed et al. (2016). Briefly, 1 g of each solid sample was pre-enriched in 9 mL of buffered peptone water (Fluka Biochemika, Steinheim, Germany), while each swab stick with its sample was inoculated into 10 mL buffered peptone water, and these were incubated at 37 °C for 18 h – 24 h; 1 mL of overnight buffered peptone water culture of each sample was then enriched in 9 mL each of selenite F broth (Oxoid Ltd, Hampshire, United Kingdom [UK]) and in Rappaport-Vassiliadis broth (Oxoid Ltd, Hampshire, UK) incubated for 18 h – 24 h at 37 °C and 42°C, respectively.

The procedures used for the isolation of *Salmonella* from the samples were described earlier (Ahmed et al. 2017; Raufu et al. 2013). Briefly, the samples on selective broths (selenite F and Rappaport-Vassiliadis) were sub-cultured onto xylose lysine deoxycholate (XLD) agar (Oxoid Ltd, Hampshire, UK) and *Salmonella–Shigella* agar (Oxoid Ltd, Hampshire, UK) and were incubated aerobically at 37 °C for 24 h. All colonies suggestive of *Salmonella* on the selective agars were purified on blood agar plates (Oxoid Ltd, Hampshire, UK) at 37 °C for 24 h and later subjected to biochemical tests, including triple sugar iron, urease, citrate, methyl red, indole, Voges–Proskauer, ONPG (ortho-nitrophenyl galactosidase), amino acid (lysine and arginine) decarboxylase, catalase and motility (Cheesbrough 2002; Perilla 2003).

### Serotyping

Serotyping of all the presumptive *Salmonella* isolates from biochemical tests was performed at the WHO National *Salmonella* and *Shigella* Center, Bangkok, Thailand, on the basis of the reaction with somatic (O), flagellar (H) and capsular (Vi) hyperimmune sera (S & A Reagents Laboratory, Ltd., Bangkok, Thailand). The serotypes were assigned according to the Kauffmann–White scheme (Popoff & Minor 2007) as previously described (Raufu et al. 2013).

### Antimicrobial susceptibility testing

Antimicrobial susceptibility testing was carried out on the serotyped *Salmonella* serovars using the disc diffusion method according to the Clinical and Laboratory Standards Institute (CLSI 2016) by culturing on the Mueller–Hinton agar (Oxoid Ltd, Hampshire, UK). The antibiotic impregnated discs utilised (Oxoid Ltd, Hampshire, UK) include ampicillin (10 *µ*g), compound sulphonamide (300 *µ*g), gentamicin (10 *µ*g), ciprofloxacin (5 *µ*g), chloramphenicol (30 *µ*g), ceftazidime (30 *µ*g), cefotaxime (30 *µ*g), neomycin (30 *µ*g), nalidixic acid (30 *µ*g), streptomycin (10 *µ*g) and tetracycline (30 *µ*g). The zones of inhibition were measured and interpreted as recommended by the CLSI (2016). The test was performed by emulsifying a well-isolated colony of the isolate onto 5 mL of normal saline in a test tube. The turbidity of the inoculum was then compared with 0.5% McFarland standard against a nephelometer. The inoculum with turbidity of 0.5% McFarland was poured and evenly spread on the Mueller–Hinton agar (Oxoid Ltd, Hampshire, UK). Excess inoculum was discarded after 2–3 minutes (min). The plates were left to dry after which antimicrobials were applied using a disc dispenser (Oxoid Ltd, Hampshire, UK). The plates were then incubated aerobically at 37 °C for 18 h using *Escherichia coli* ATCC 25922 (CCM 3954) as a control strain.

### Statistical analysis

Prevalence was calculated by dividing the number of samples positive for *Salmonella* by the total number of samples processed. The significance (*p* < 0.05) of differences between isolation rates of *Salmonella* from various sources was calculated using a chi-square test for independent proportion.

### Ethical considerations

Ethical approval was obtained from the University of Ilorin, Faculty of Veterinary Medicine Ethical Review Committee, with approval code number FVER/001/2016.

## Results

### Isolation rate of salmonellae from different sample types

This study showed that out of the 900 samples collected from three different sample types comprising of the poultry environment, dead and live birds, 58 were positive for *Salmonella* giving an overall isolation rate of 6.4%. The isolation rate of *Salmonella* serovar varied among the farms ranging from 4% in farms 7 and 8, to 16% in farm 9 (Table 2). The highest frequency of the isolation of *Salmonella* was obtained from live birds (8%), while the least was from dead birds (4.7%). Among the different sampling units in the poultry environment, feed from feeding trough recorded the highest rate of isolation of 11.1%, while the lowest rate was obtained from water in both drinker and reservoir (4.4% each). Considering different sampling units in dead birds, the highest frequency of isolation was obtained from the liver (8.3%; Table 3). The isolation rate was only statistically significant (*p* < 0.05) in live birds.

**TABLE 2:** Number of samples collected and *Salmonella* isolation rate per farm.

Farm no.	No. of samples collected	Positive samples	
		*N*	%
F1	100	5	5.0
F2	100	8	8.0
F3	100	6	7.0
F4	100	5	5.0
F5	100	5	5.0
F6	100	5	5.0
F7	100	4	4.0
F8	100	4	4.0
F9	100	16	16.0
**Total**	**900**	**58**	**6.4**

**TABLE 3:** Isolation rate of Salmonellae from different sample types.

Farm no.	Positive samples/farm																								
	Poultry environment											Dead birds											Live birds		
	Litter		Feed				Water				*p*	Liver		Spleen		Ovarian		Caecum		Heart		*p*	Cloacal swabs (*n* = 35f)		*p*
			F		S		D		R																
	*N*	%	*N*	%	*N*	%	*N*	%	*N*	%		*N*	%	*N*	%	*N*	%	*N*	%	*N*	%		*N*	%	
F1	0	0	1	20	0	0	0	0	0	0		0	0	0	0	1	13	0	0	0	0		3	9	
F2	1	20	0	0	1	20	1	20	1	20		1	13	1	13	0	0	0	0	0	0		2	6	
F3	1	20	1	20	0	0	0	0	0	0		1	13	1	13	1	13	0	0	1	13		0	0	
F4	0	0	0	0	0	0	0	0	0	0		2	25	2	25	0	0	1	13	0	0		0	0	
F5	1	20	0	0	0	0	1	20	0	0		1	13	0	0	0	0	0	0	2	25		0	0	
F6	0	0	1	20	0	0	0	0	0	0		1	13	0	0	0	0	0	0	0	0		3	9	
F7	0	0	0	0	2	40	0	0	0	0		0	0	0	0	0	0	1	13	0	0		1	3	
F8	0	0	0	0	0	0	0	0	0	0		0	0	0	0	0	0	0	0	0	0		4	11	
F9	0	0	2	40	1	20	0	0	1	20		0	0	0	0	0	0	0	0	0	0		12	34	
Subtotal	3	7	5	11	4	9	2	4	2	4.4	0.22	6	8	4	5.6	2	2.8	2	2.8	3	4	0.09	25	8	0.05*
**Total/source**	**16(7.1)**											**17(4.7)**											**25(8.0)**		

F, feeding trough; S, feed store; D, drinking trough; R, water reservoir.

* , *p* < 0.05.

### *Salmonella* serovars distribution among the poultry farms

The 58 *Salmonella* isolates revealed 13 different serovars which included *Salmonella* Agama (*S.* Agama), *S.* Albany, *S*. Colindale, *S. enterica* ser. 4.5.12:i: -, *S. enterica* ser. 4.12.27:z: -, *S. enterica* ser. 6.7:d:-, *S. enterica* ser. 45:d: 1.7, *S.* Istanbul, *S.* Larochelle, *S.* Muenster, *S.* Nigeria, *S.* Orion and *S.* Typhimurium. *Salmonella enterica* ser. 6.7:d:- was the most frequently isolated, accounting for (17/58) 29% of all the serovars. *Salmonella* Agama accounted for (16/58) 28%, while *S.* Typhimurium accounted for (9/58) 16% of the *Salmonella* serovars. *Salmonella* Agama was isolated from all the farms, while *S. enterica* ser. 6.7:d:- was isolated from six of the nine farms sampled (Table 4).

**TABLE 4:** Occurrence of *Salmonella* serovars in different poultry farms in Kwara State.

Serovars	Serovars/farm																		Total	
	F1		F2		F3		F4		F5		F6		F7		F8		F9			
	*N*	%	*N*	%	*N*	%	*N*	%	*N*	%	*N*	%	*N*	%	*N*	%	*N*	%	*N*	%
*S.* Agama	1	6	1	6	1	6	1	6	2	13	5	31	1	6	1	6	3	19	16	28
*S.* Albany	0	0	0	0	0	0	0	0	1	100	0	0	0	0	0	0	0	0	1	2
*S*. Colindale	0	0	0	0	0	0	0	0	0	0	0	0	1	100	0	0	0	0	1	2
*S. enterica* ser. 4,5,12 :i:-	0	0	0	0	0	0	0	0	0	0	0	0	1	100	0	0	0	0	1	2
*S. enterica* ser. 4,12,27:z:-	0	0	0	0	0	0	0	0	0	0	0	0	0	0	0	0	1	100	1	2
*S. enterica* ser. 6,7:d :-	4	22	5	28	1	6	0	0	1	6	0	0	0	0	2	11	4	22	17	29
*S. enterica* ser. 45: d :1,7	0	0	0	0	2	40	0	0	0	0	0	0	0	0	0	0	3	60	5	9
*S.* Istanbul	0	0	0	0	1	100	0	0	0	0	0	0	0	0	0	0	0	0	1	2
*S.* Larochelle	0	0	0	0	0	0	1	50	0	0	0	0	0	0	0	0	1	50	2	3
*S.* Muenster	0	0	2	100	0	0	0	0	0	0	0	0	0	0	0	0	0	0	2	3
*S.* Nigeria	0	0	0	0	0	0	0	0	0	0	0	0	1	100	0	0	0	0	1	2
*S.* Orion	0	0	0	0	1	100	0	0	0	0	0	0	0	0	0	0	0	0	1	2
*S.* Typhimurium	0	0	0	0	0	0	3	33	1	11	0	0	0	0	1	11	4	44	9	16
**Total**	**5**	**9**	**8**	**14**	**6**	**10**	**5**	**9**	**5**	**9**	**5**	**9**	**4**	**7**	**4**	**7**	**16**	**28**	**58**	**100**

**FIGURE 2:**
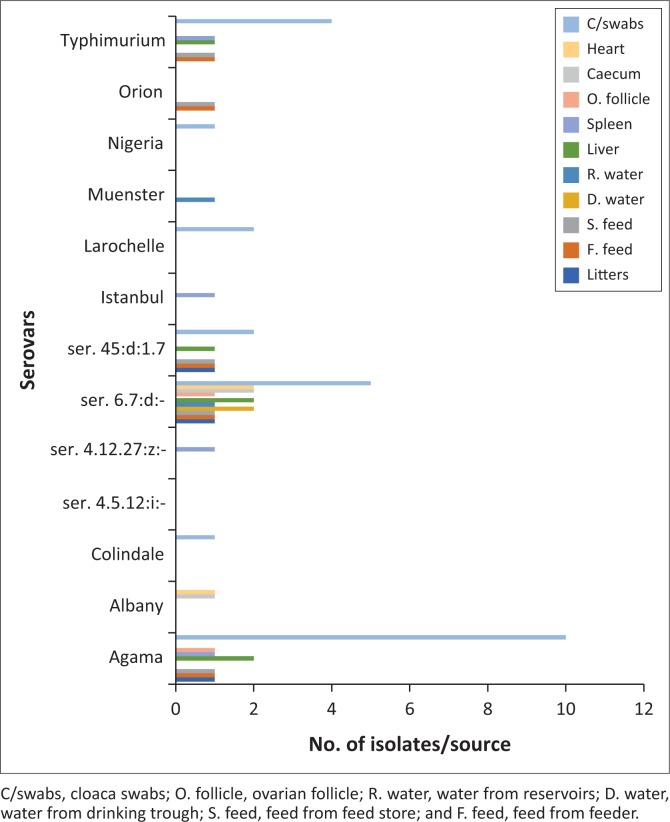
Frequency of *Salmonella* serovars isolated from different sites.

Eight different serovars were isolated from dead birds representing the highest number of serovars from a single source. The majority of the most prevalent serovars were obtained from multiple sources. *Salmonella enterica* ser. 6.7:d:- was isolated from all the samples except from liver and caecum. *Salmonella* Agama was obtained from the poultry environment (feed and water), dead birds (liver, spleen and ovarian follicle) and apparently healthy birds (cloaca swabs); while *S.* Typhimurium was isolated from feeds, dead birds (liver, spleen and caecum) and live birds (cloaca swabs) (Figure 2).

### Antimicrobial susceptibility testing

In general, a high percentage of resistance to the tested antimicrobials was observed across all the serovars. All the serovars (100%) were resistant to ampicillin, ceftazidime and cefotaxime. The *Salmonella* Albany isolated was resistant to all antimicrobials used except chloramphenicol, neomycin, compound sulphonamides and tetracycline. *Salmonella* Nigeria and *Salmonella enterica* ser. 4.5.12:i:- were resistant to all the antimicrobials except neomycin. All the isolates showed high resistance to ciprofloxacin and nalidixic acid (≥50%) except *Salmonella* Muenster, which was susceptible to ciprofloxacin, and *Salmonella* Colindale, which was susceptible to the two antimicrobials. *Salmonella enterica* subspecies *enterica* ser. 4.12.27:z:- showed resistance to all of the antimicrobial agents used (Table 5).

**TABLE 5:** Antimicrobial profiles of *Salmonella* serovars isolated from different poultry farms in Kwara State.

Serovars	No. of positive	Isolates showing resistance to the antimicrobials																					
		AMP		C		CAZ		CIP		CN		CTX		N		NA		S		S		TE	
		*N*	%	*N*	%	*N*	%	*N*	%	*N*	%	*N*	%	*N*	%	*N*	%	*N*	%	*N*	%	*N*	%
*S.* Agama	16	16	100	8	50	16	100	8	50	8	50	16	100	2	13	11	69	7	44	11	69	11	69
*S.* Albany	1	1	100	0	0	1	100	1	100	1	100	1	100	0	0	1	100	0	0	1	100	0	0
*S.* Colindale	1	1	100	0	0	1	100	1	100	0	0	1	100	1	100	0	0	0	0	0	0	0	0
*S.* enterica ser. 4,5,12 : i :-	1	1	100	1	100	1	100	1	100	1	100	1	100	0	0	1	100	1	100	1	100	1	100
*S.* enterica ser. 4,12,27 :z :-	1	1	100	1	100	1	100	1	100	1	100	1	100	1	100	1	100	1	100	1	100	1	100
*S.* enterica ser. 6,7: d :-	17	17	100	8	47	17	100	11	65	9	53	17	100	5	35	11	65	8	47	11	65	9	53
*S. enterica* ser. 45: d : 1,7	5	5	100	1	20	5	100	3	60	2	40	5	100	1	20	1	20	1	20	1	20	2	40
*S.* Istanbul	1	1	100	0	0	1	100	1	100	1	100	1	100	0	0	1	100	0	0	0	0	0	0
*S.* Larochelle	2	2	100	1	50	2	100	2	100	1	50	2	100	1	50	2	100	2	100	1	50	2	100
*S.* Muenster	2	2	100	2	100	2	100	0	0	2	100	2	100	2	100	1	50	1	50	2	100	1	50
*S.* Nigeria	1	1	100	1	100	1	100	1	100	1	100	1	100	0	0	1	100	1	100	1	100	1	100
*S.* Orion	1	1	100	0	0	1	100	1	100	0	0	1	100	0	0	1	100	0	0	0	0	1	100
*S.* Typhimurium	9	9	100	5	56	9	100	6	67	5	56	9	100	6	67	3	33	6	67	7	78	7	78

AMP, ampicillin (10 *μ*g); C, chloramphenicol (30 *μ*g); CAZ, ceftazidime (30 *μ*g); CIP, ciprofloxacin (5 *μ*g); CN, gentamycin (30 *μ*g); CTX, cefotaxime (30 *μ*g); N, neomycin (30 *μ*g); NA, nalidixic acid (30 *μ*g); S, compound sulphonamides (300 *μ*g); S, streptomycin (10 *μ*g); TE, tetracycline (30 *μ*g).

## Discussion

*Salmonella* serovars were isolated from all of the three sample types examined. *Salmonella* is an important zoonotic pathogen and its occurrence in animals poses a continuous threat to man (Muragkar et al. 2005). The isolation rate of *Salmonella* from this study corroborated a similar study from Maiduguri, northeastern Nigeria, with a rate of 7% (Raufu et al. 2013), and Ibadan, southwestern Nigeria, with a rate of 10% (Fashae et al. 2010). A higher rate (37%) of *Salmonella* contamination on broiler farms had been reported from Algeria (Elgroud et al. 2009), thus suggesting chickens and poultry environments as important reservoirs of *Salmonella* in Nigeria.

The overall frequency of isolation of *Salmonella* from the cloacae of birds was 8% in this study; this is in agreement with the range (4% – 12%) reported in Algeria by Ammar et al. (2010). However, it was higher than 0.9% reported in Trinidad and Tobago (Adesiyun et al. 2014). Swabs of litter from poultry farms had a high frequency (6.7%) of *Salmonella* contamination, which indicates that freshly laid eggs in the studied farms have a higher risk of being contaminated by *Salmonella.* The overall isolation rate of *Salmonella* from the poultry environment in this study (7.1%) was within the range of 0.95% to 33.3% reported by other researchers (Adesiyun et al. 2014; Shirota et al. 2012). Feeds, particularly in a deep litter management system, can be a source of contamination to eggs laid in the litter (Adesiyun et al. 2014; Shirota et al. 2012). The isolation rate of *Salmonella* from feed samples in this study was high (10%) and might not be unrelated to feed formulation components such as blood meal, fish meal, bone meal, egg shells (animal), groundnut cake and soya bean cake which are, in most cases, not stored properly or are unhygienically processed, thus serving as a source of contamination of feed. More importantly, the weather in the study area is usually warm and humid, and *Salmonella* organisms can, under these circumstances, multiply in the feed, especially during storage on the farms (Jones & Richardson 2004).

In this study, the predominant serovars were *Salmonella enterica* ser. 6.7:d:- (29%), *S.* Agama (28%) and *S*. Typhimurium (16%). *Salmonella enterica* ser. 6.7:d:- has similar antigenic formula with *Salmonella* Kivu (6.7:d:1,6). *Salmonella* Kivu was first characterised in 1961 in the Congo (Van Oye, Van Ros & Herman 1961) and was reported to cause human sporadic diarrhoea in Durban, South Africa (Govinden et al. 2008). *Salmonella* Kivu had also been reported albeit at a lower level (0.62%) in poultry processing environments in Malaysia (Nidaullah et al. 2017). To the best of our knowledge, no study has been published to date on whether there is a genotypic relationship between the serovar Kivu and the one (6.7:d:-) isolated in this study. *Salmonella* Typhimurium is also a common serovar in chickens and has been reported by other workers in Nigeria (Fasure, Deji-Agboola & Akinyemi 2013; Orji et al. 2005). It has also been documented from poultry in Trinidad and Tobago and Algeria (Adesiyun et al. 2014; Jakirul et al. 2016). There has been report of an epidemic increase in the prevalence of *S*. Typhimurium which has been linked to the circulation of a particular multilocus sequence typing clone, ST313, in sub-Saharan African countries (Kingsley et al. 2009); however, it has not been determined if the *S*. Typhimurium ST313 clone has spread to Kwara State, Nigeria. Further study(ies) will reveal if these isolates belong to the previously described clone of phage type U282 in Nigeria (Ojeniyi & Montefiore 1986). *Salmonella* Agama was characterised in 1956 as a new serotype of *Salmonella enterica* from faeces of the Agama lizard (*Agama agama*) in Nigeria (Collard & Montefiore 1957). Subsequently, *S.* Agama was isolated from geckos and mammals in Africa (Collard & Sen 1960; Oboegbulem & Okoronkwo 1990; Orji et al. 2005) and the United Kingdom (Davies & Breslin 2004; Wilson et al. 2003). It was also reported as a contaminant in poultry feed mills in the United Kingdom (Davies & Wales 2010). Human infections with *S.* Agama were reported in Nigeria and related to the lizards as a possible reservoir (Collard & Sen 1960). It was also reported to have caused traveller’s diarrhoea in Gabon (Bélard, Kist & Ramharter 2007) and France, in a 9-month-old child with fever and diarrhoea (Appas, Kieffer & Sigwalt 1966). It was also incriminated in neonatal meningitis in the United Kingdom (Paul et al. 2015) and human sporadic diarrhoea in Okinawa, Japan (Jun et al. 2006). Although *S*. Agama was reported recently in Nigeria from faecal droppings and poultry feeds, it was not among the major serovars (3.7%) isolated in the study of Idowu et al. (2017). *Salmonella* Agama was isolated from all the sampling units except water in this study. This is important to public health as the birds and poultry environment colonised with *Salmonella* can be sources of infection to man. The occurrence of *S.* Agama in chickens and the poultry environment in the study area might be because of the abundance of Agama lizards around all habitations, including the poultry pen and the poultry environment.

The high level of resistance to most of the antimicrobials tested in this study, especially nalidixic acid and ciprofloxacin, is worrisome because fluoroquinolones are used strategically in the treatment of salmonellosis. This resistance may be because of indiscriminate use of antimicrobials at recommended doses or at subtherapeutic doses in feed as growth promoters, and as chemotherapeutic agents to control epizootics on the farms; however, it is important to inquire the types of antimicrobials the farmers administer to their birds either as prophylaxis or therapeutics before studying the antimicrobials resistance in future studies. The lack of policy to control the use of antimicrobials, especially fluoroquinolones, including ciprofloxacin, enrofloxacin and ofloxacin in poultry in Nigeria, may have contributed to the rapid spread of resistance in the poultry industries (Parry & Threlfall 2008). These findings agreed with the report of Fashae et al. (2010) which equally reported a high level of resistance to nalidixic acid and reduced susceptibility to ciprofloxacin. The resistance to cephalosporins (ceftazidime and cefotaxime) is in agreement with Vincent et al. (2008), Agada et al. (2014) and Ahmed et al. (2016). This is worrisome, in view of the high level of resistance observed for all of the *Salmonella* serovars isolated in this study. Cephalosporins are major antimicrobials used to treat serious *Salmonella* infections in humans. However, their effectiveness is being compromised by the emergence of extended-spectrum beta-lactamases (ESBLs) and plasmid-mediated cephalosporinases (Vincent et al. 2008). The low level of resistance by most of the isolates to neomycin might be because of the fact that the farmers in the study area have neglected this drug and opted for some alternate effective antimicrobials like ciprofloxacin. *Salmonella* Agama, which is of zoonotic significance, was one of the most prevalent serovars in this study and showed a high level of resistance to most of the commonly used antimicrobials. These observations call for regulation of antibiotic usage in Nigeria to ameliorate the spread of resistance to antimicrobials.

## Conclusion

This study established the presence of *Salmonella* in poultry farms and their environment in Kwara State. In addition, this study reported the occurrence of rare serovars that are of zoonotic importance and can be of global importance as a result of travels, transhumance, and the animal and food products trade. This study also highlighted the diffuse prevalence of resistance to critically important antimicrobials like fluoroquinolones and cephalosporins.
